# Hematological abnormalities and associated factors among adult HIV/AIDS patients on antiretroviral therapy at Hiwot Fana Comprehensive Specialized Hospital: A cross sectional study

**DOI:** 10.1097/MD.0000000000046223

**Published:** 2026-05-12

**Authors:** Beza Sileshi, Haftu Asmerom, Kabtamu Gemechu, Gada Diba, Shambel Mekonnen, Mesay Arkew

**Affiliations:** a Department of Medical Laboratory Science, School of Medical Laboratory Sciences, College of Health and Medical Sciences, Haramaya University, Harar, Ethiopia.

**Keywords:** associated factors, Eastern Ethiopia, hematological abnormalities, HIV/AIDS

## Abstract

Hematological abnormalities are common in individuals with human immunodeficiency virus (HIV) infection and can have significant clinical implications. These abnormalities can affect various components of the blood, including red blood cells, white blood cells, and platelets. However, screening and follow up of HIV positive patients for these abnormalities is not well reported in Ethiopia, specifically in Harar. Therefore, this study is aimed to determine prevalence of hematological abnormalities and associated factors among Adult HIV infected patients on antiretroviral therapy at the Antiretroviral clinic of Hiwot Fana Comprehensive Specialized Hospital, Eastern Ethiopia, from September 2, October 30, 2024. A hospital based cross-sectional study was conducted by involving 300 study participants using convenient sampling technique. Data related to socio-demographic, clinical and dietary variables were collected using structured questionnaires. All phase of quality assurance was maintained. Four milliliters of venous blood were collected from each study participant and complete blood count was determined using unicell DxH 800 hematological analyzer. Logistic regression was conducted to assess the association between hematological abnormalities and independent variables. *P* < .05 was considered statically significant. The overall prevalence of hematological abnormality among HIV/acquired immunodeficiency syndrome (AIDS) patients were 26.33%. Whereas prevalence of anemia, leucopenia and thrombocytopenia in this study were 16.7%, 4.33%, and 5.3%, respectively. HIV/AIDS patients with underweight BMI (AOR = 3.94, 95% CI: 1.08–14.36), male gender (AOR = 2.71, 95% CI: 1.35–5.44), HIV patients who have lived with HIV infections for more than 12 years (AOR = 2.77, 95% CI: 1.43–5.39) and HIV patients who have a monthly income of 1500 to 5000 Ethiopian birrs (AOR = 4.38, 95% CI: 1.19–16.1) were significantly associated with anemia. hematological abnormality was found to be a moderate public health problem among anti-retroviral therapy attendants in the current study area. Also, early detection and intervention among, males, patients having body mass index < 18.5 kg/m^2^, patients having low monthly income and patients who lived with HIV for more than 12 years is vital to reduce the magnitude of anemia and its consequences.

## 1. Introduction

Human immunodeficiency virus (HIV) is an infection that attacks the body’s immune system, with acquired immunodeficiency syndrome (AIDS) being the most advanced stage. HIV targets white blood cells (WBCs), weakening the immune system, making it easier to contract diseases like tuberculosis (TB), infections, and some cancers.^[1]^ Anti-retroviral therapy (ART) is a treatment for HIV-infected individuals using anti-HIV drugs, often called “highly active antiretroviral therapy” (HAART). Antiretroviral therapy prevents HIV transmission, enhances quality of life, and lowers death and morbidity rates.^[2]^ HIV-positive people frequently experience hematological abnormalities such as anemia, leukopenia, and thrombocytopenia, which can have serious clinical repercussions.^[3]^ Although the precise processes underlying these changes remain unclear, it is thought that inflammation, direct viral impacts on the bone marrow, and chronic immunological activation are to blame.^[4]^ Management of hematological abnormalities typically involves treating the underlying cause, such as initiating antiretroviral therapy to control viral replication and reduce immune activation. In some cases, specific interventions may be required, such as blood transfusions for severe anemia or growth factors to stimulate WBC or platelet production.^[5]^

HIV remains a significant global problem, particularly in sub-Saharan Africa, with approximately 39 million people living with the virus as of 2022. Ethiopia, one of the largest populations in the region, has one of the largest HIV populations in the area. According to the Joint United Nations Programmed on HIV/AIDS, as of 2020, an estimated 690,000 people were living with HIV in Ethiopia.^[6]^ Common clinicopathological characteristics of HIV infection include hematological abnormalities, which can serve as a reliable indicator of morbidity and death. Anemia and granulocytopenia occur in a considerable percentage of asymptomatic carriers and individuals with AIDS, and their incidence and severity increase as the disease progresses.^[7]^ Limited access to healthcare services and resources in Ethiopia exacerbates the problem, leading to undiagnosed or untreated conditions. Factors contributing to hematological alterations in Ethiopia may include nutritional deficiencies, co-infections with opportunistic pathogens, and limited availability of certain antiretroviral medications.

This study evaluates the hematological profile of HIV-positive individuals in Ethiopia, offering important insights for better HIV care. It closes knowledge gaps on hematological changes in HIV patients receiving HAART, assists hospital management in planning medications and supplies, and helps clinicians create the best strategies. It also acts as a resource for upcoming studies.

## 2. Materials and methods

### 2.1. Study design, area, and period

The study was conducted in Harar city, located 526 km far from Addis Ababa, the capital city of the country, in the Harari National Regional State, Eastern Ethiopia. According to the current regional health bureau profile, the Harari region has a total of 7 hospitals, including 4 governmental hospitals, 2 private hospitals, one non-government (Fistula) hospital, 8 health centers, and 29 private clinics. This study was conducted at Hiwot Fana Comprehensive Specialized Hospital (HFCSH). Hiwot Fana Hospital is a comprehensive specialized university hospital located in Harar town, and it currently provides various services for more than 5.8 million people in the catchment area. It has more than 34 outpatient clinics, 15 inpatient departments and a separate emergency and trauma center. It also has an Oncology center functional since 2020 that provides radiation therapy as one of the 4 centers in Ethiopia. ART services are among the many services provided by the hospital. According to the HFCSH report, there are 1964 HIV/AIDS patients getting service in the ART clinic of the hospital.^[8]^ The study period was from September 2 to October 3, 2024.

#### 2.1.1. Population

All adult HIV/AIDS patients who follow up at the ART clinic of HFCSH were considered as the source population. All adult HIV/AIDS patients who followed up at ART clinic of HFCSH and who visited the ART clinic during data collection time were included. On the other hand, participants who are pregnant, have chronic diseases like cardiac, renal, and liver diseases, those who take anticoagulants, smokers, alcoholics, and HIV/AIDS patients with recent vitamin supplements were excluded. The participants were excluded following a critical review of their medical records and face-to-face interviews.

#### 2.1.2. Sample size determination and sampling technique

The sample size was calculated using single population proportion formula by considering the previous prevalence of anemia (23.1%) reported from Jimma University specialized hospital using a 95% confidence level, margin of error of 5%, and 10% non-response rate.^[9]^ A total of 300 HIV/AIDS patients participated in this study. A convenient sampling technique was used in consecutive order until the calculated sample size was reached.

#### 2.1.3. Data collection methods

##### 2.1.3.1. Socio-demographic, clinical, and anthropometric data collection procedure

Before data collection, the data collector (trained nurse) selected patients who fulfilled inclusion criteria and took informed consent. Data related to sociodemographic and nutritional variables were collected using a structured questionnaire through face-to-face interviews. Data related to clinical variables and medication was collected from medical record reviews and interviews. After the interview, medical record review, and anthropometric measurement (height and weight) were taken, the study subjects were sent to the laboratory where a blood sample was taken for determination of hematological parameters and stool for intestinal parasite examination.

##### 2.1.3.2. Laboratory sample collection and analysis

Four milliliters of venous blood were collected from each study participant and by laboratory professionals under aseptic conditions and transferred to ethelene diamine tetra acetic acid (EDTA) anticoagulant tubes. The blood sample was collected in EDTA anticoagulant tubes. The blood sample collected in the EDTA tube was gently mixed by inverting the tube 6 to 8 times to prevent clotting and used for hematological tests. Complete blood count was analyzed by Unicel DxH 800 hematology analyzer. About one gram (pea size) of fresh fecal spacemen was collected using a labeled, clean, dry, leak-proof container. Intestinal parasites were examined by direct wet mount and formol-ether concentration technique.

##### 2.1.3.3. Operational definitions

Hematological abnormalities: having any hematological change including anemia, thrombocytopenia, leukopenia.

Anemia: is a condition in which the number of red blood cells (RBCs) or the hemoglobin concentration within them is lower than normal. To classify severity of anemia we used World Health Organization (WHO) classification criteria briefly Hemoglobin value in g/L as mild, moderate and severe depending on sex and age. See details of anemia classification in Table 1 below.

**Table 1:** Anemia diagnosis criteria according to World Health Organization.

Population	Non anemia	Mild	Moderate	Severe
Non pregnant women	120 or higher	110–119	80–109	<80
Pregnant	110 or higher	110–109	70–99	<70
Men	130 or higher	110–129	80–129	<80

Leukopenia: is a reduction in the circulating WBC count to <4000/mcL (<4 × 10^9^/L) thrombocytopenia: a platelet count that falls below the lower limit of normal, i.e., 150,000/µL (for adults) is defined as thrombocytopenia.

Cytopenia: is a condition in which there is a lower-than-normal number of blood cells that encompasses anemia, neutropenia, thrombocytopenia, and/or pancytopenia.

Viral load refers: to the amount of virus in an infected person’s blood, this expressed as the number of viral particles in each milliliter of blood.

Recent vitamin supplement: is operationally defined as someone who takes vitamin within the past 2 weeks.

Nutritional counseling:is a process that helps people improve their nutrition and health by hite providing information, education, and personalized recommendations.

Bicytopenia: a medical condition characterized by a reduction chatcterized by reduction in the counts of any 2 of 3 main blood cell linages RBCs (anemia), WBCs (lukopenia) and plateletes (thrombocytopenia).

##### 2.1.3.4. Data quality assurance and management

To ensure the quality of data, all phases of quality assurance were maintained. The English version of the questionnaire was translated into the local language (Amharic and Afaan Oromo) and re-translated to English. A pretest was conducted on 5% of the sample size at Jugal Hospital before actual data collection and training was given to data collectors on the objective of the study, confidentiality of information, and the data collection process. Standard operating procedures were strictly followed for each laboratory activity. All laboratory reagents were checked for their expiry date before use. Daily quality control was performed to ensure the performance of the hematological analyzer. All activities were supervised by a senior medical laboratory professional.

##### 2.1.3.5. Data analysis

The collected data was entered into Epi Data version 4.6 and then exported to Statistical Products and Service Solutions Version 27 for analysis. Descriptive statistics was computed and summarized in figures, tables, and text with frequencies, mean, or standard deviations. Bivariate and multivariable logistic regression analysis was used to identify the association between independent variables and outcome variable. The model goodness of fit was tested by the Hosmer–Lemeshow test and multicollinearity was checked by variance inflation factor. The association between anemia and the independent variables was reported as an odds ratio with 95% CI. The variables having a *P*-value < .25 in the bivariable analysis were entered into the multivariable analysis and a *P*-value of < .05 was considered as statistically significant.

##### 2.1.3.6. Ethical considerations

Ethical clearance was obtained from the Institutional Health Research Ethics Review Committee of Haramaya University College of Health and Medical Sciences. The reference no for this study was IHRERC/204/2024. Written and signed consent was obtained from the study participants, by clearly explaining the purpose, procedures, potential risks, and benefits, also assuring them the confidentiality and voluntary nature of participation in the study. Information was provided in a language and format that was easily understandable to the participants. Participation in the study was voluntary, participants had the right to refuse or withdraw from the study at any time without facing any negative consequences. There were no coercive measures or undue influence to encourage participation.

### 2.2. Result

#### 2.2.1. Sociodemographic characteristics of study participants

A total of 300 study participants were enrolled in this study. The age of the study participants ranged from 17 to 78 years. The majority (50.6%) of the study participants were more than 40 years of age. Most of the study participants 217 (72.3%) were females, and 274 (91.3%) of the study participants were from urban areas. Most of the study participants were at primary school (49.7%) and married (58%) (Table 2).

**Table 2:** Sociodemographic status of study participants at Hiwot Fana Comprehensive Specialized Hospital, Harar, Eastern Ethiopia 2024 (n = 300).

Variable	Category	Frequency	Percent
Sex	Male	83	44.7
	Female	217	72.3
Age group	≥40	152	50.6
	<40	148	49.4
Residence	Urban	274	91.3
	Rural	26	9.7
Marital status	Married	174	58
	Single	36	12
	Divorced	53	17.7
	Widowed	37	12.4
Educational status	Unable to read and write	37	12.3
	Elementary	149	49.7
	Secondary	91	30.3
	Higher education	23	7.7
Income status	500–1500	82	27.3
	1500–5000	197	65.7
	>5000	21	7

#### 2.2.2. Clinical characteristics of the study participants

Among the study participants 179 (59.7) and 121 (6.7%) of them have been with HIV for less than or equal to 12 years and above 12 years, respectively. Most of study participants 274 (83.3%) take non zidovudine containing ART regimen.

Among the respondents, 260 (86.7%) of them had switched drug regimens and the rest 40 (13.3%) had not. Most of the study participants were on WHO Stage III and IV 173 (57.7%). There were 13 (3.33%) opportunistic infections and among those 3 were TB 2 were Candidiasis and the rest 8 are salmonella. Among those 3 TB cases, all of them were taking first-line anti-TB drugs (Tables 3 and 5).

**Table 3:** Clinical characteristics of study participants at Hiwot Fana Comprehensive Specialized Hospital, Harar, Eastern Ethiopia 2024 (n = 300).

Variable	Category	Frequency	Percent
WHOstage	Stage 1	45	15
	Stage2	82	27.3
	Stage 3and 4	173	57.7
Duration of HIV infection	≤12 yr	179	59.7
	>12 yr	121	40.3
Opportunistic infection	Yes	13	3.3
	No	287	96.7
Types of opportunistic infection	TB	3	30
	Candidiasis	2	20
	Salmonella	8	20
Intestinal parasite	Yes	43	14.3
	No	257	85.7
Species of intestinal parasite	E. histolytica	18	41.8
	G. lamblia	25	58.1
Viral load	Detected	13	14.3
	Undetected	287	85.7

HIV = human immunodeficiency virus, TB = tuberculosis.

**Table 4:** Nutritional characteristics of study participants at Hiwot Fana Comprehensive Specialized Hospital, Harar Eastern Ethiopia 2024 (n = 300).

Variable	Category	Frequency	Percent
BMI	UNDERWEIGHT	24	8
	NORMAL	183	61
	OVERWEIGHT	71	23.7
	OBESE	22	7.3
Nutritional support	Yes	2	0.66
	No	298	99.33
Frequency of eating	Twice	68	22.7
	Three times	228	76
	Four times	4	1.3
Breakfast	Yes	28	9.3
	No	272	90.7
Lunch	Yes	40	13.3
	No	260	86.7
Dinner	Yes	0	0
	No	300	100

#### 2.2.3. Nutritional characteristics of participants

Among the study participants 24 (8%), 183 (61%), 71 (23.7%), and 22 (7.3%) of them are underweight, normal, overweight, and obese respectively. only 2 of them had nutritional support. 68 (22.7%), 228 (76%), and 4 (1.3%) of them have daily eating frequency of 2 times, 3 times and 4 times respectively. 28 (9.3%), 40 (13.3%), and none of them skipped their breakfast, lunch, and dinner respectively (Table 4).

**Table 5:** Medication related characteristics of study participants at Hiwot Fana Comprehensive Specialized Hospital, Harar, Eastern Ethiopia 2024 (n = 300).

Variable	Category	Frequency	Percent
Current regimen	ZDV containing	26	16.7
	Non ZDV containing	274	83.3
Previous regimen	ZDV containing	82	27.3
	Non ZDV containing	218	72.7
Switch regimen	Yes	260	86.7
	No	40	13.3
Anti TB drug	Yes	3	1
	No	297	99

PLT = platelet, TB = tuberculosis, ZDV = zidovudine.

#### 2.2.4. Hematological profile of study participants

The mean plus SD of the hematological profiles are WBC count of 6.49 ± 1.83, RBC count of 4.48 ± 0.41, and platelet count of 264.82 ± 74.58 and see the details below in Table 6.

**Table 6:** Hematological profile of study participants at Hiwot Fana Comprehensive Specialized Hospital, Harar, Eastern Ethiopia 2024 (n = 300).

Variable	Mean	SD
WBC count (×10^3^/µL)	6.49	1.83
RBC count (×10^6^/µL)	4.48	0.41
HGB (g/dL)	13.81	1.40
HCT (%)	39.14	3.77
MCV (fL)	91.22	6.90
MCH (Pg)	31.94	2.47
MCHC (%)	34.32	1.81
RDW-CV (%)	13.68	1.36
Neutrophil (×10^3^/µL)	3.21	1.37
Eosinophil (×10^3^/µL) (%)	0.28	0.16
Basophil (×10^3^/µL)	0.03	0.11
Lymphocyte (×10^3^/µL)	2.40	0.76
Monocyte (×10^3^/µL)	0.55	0.28
PLT count (×10^3^/µL)	264.82	74.58

HCT = Hematocrit, HGB = hemoglobin, MCH = mean corpuscular hemoglobin, MCHC = mean corpuscular hemoglobin concentration, MCV = mean corpuscular volume, PLT = platelet, RBC = red blood cell, RDW-CV = red blood cell distribution width-coefficient of variation, WBC = white blood cell.

#### 2.2.5. Prevalence of hematological abnormalities

The overall prevalence of hematological abnormality among HIV/AIDS patients was 26.33%. Whereas the prevalence of anemia, leucopenia, and thrombocytopenia in this study were 16.7% (50/300), 4.33% (13/300), and 5.3% (16/300), respectively. In addition, prevalence of bi-cytopenia in this study is 2.7%.

The severity of anemia in this study was categorized as 86% mild anemia, 12% moderate anemia, and 2% severe anemia.

#### 2.2.6. Associated factors of anemia

In bivariable logistic regression, body mass index (BMI), frequency of eating, monthly income, duration of HIV infection, and sex were found to have a *P*-value less than or equal to .25 and selected as candidates for the final multivariable analysis. During the multivariable logistic regression, BMI, duration of HIV infection, sex and monthly income were significantly associated with the prevalence of anemia at a *P*-value .05 (Table 7).

**Table 7:** Multivariable analysis for factors associated with anemia among HIV/AIDS patients at Hiwot Fana Comprehensive Specialized Hospital, Harar, Eastern Ethiopia, 2024 (n = 300).

Variable	Category	Anemia		COR	*P* value	AOR	*P* value
		Yes	No				
Sex	Male	23	60	2.70 (1.44–5.05)	.002	2.71 (1.35–5.44)	.005
	Female	27	190	1		1	
BMI	Underweight	6	18	4.10 (1.23–13.64)	.022	3.94 (1.08–14.36)	.038
	Normal	37	146	3.11 (1.33–7.29)	.009	4.01 (1.63–9.85)	.002
	Overweight and obese	7	86	1		1	
Frequency of eating	2 times or less	15	53	1.59 (0.810–3.14)	.177	1.69 (0.8–3.57)	.168
	3 times or more	35	197	1		1	
Duration of HIV infection	≤12 yr	20	159	1	.002	1	.003
	>12 yr	30	91	2.62 (1.41–4.88)		2.77 (1.43–5.39)	
Monthly income (Ethiopian birr)	<1500	8	77	1.108 (.28–4.45)	.885	1.72 (0.40–7.45)	.468
	1500–5000	39	141	2.950 (.86–10.15)	.086	4.38 (1.19–16.09)	.026
	≥5000	3	32	1		1	

AIDS = acquired immunodeficiency syndrome, AOR = adjusted odds ratio, COR = crude odds ratio, HIV = human immunodeficiency virus.

The likelihood of anemia among HIV/AIDS Patients who have a BMI category of underweight were 3.94 times more likely to develop anemia (AOR = 3.94, 95% CI: 1.08–14.36) compared to those who are obese. The odds of anemia among HIV patients in terms of gender showed that males are 2.71 times more likely to develop anemia compared to females (AOR = 2.71, CI: 1.35–5.44). HIV patients who have lived with HIV infections for more than 12 years were 2.77 times more likely to have anemia compared to those who lived with HIV for <12 years (AOR = 2.77, 95% CI: 1.43–5.39). Also, HIV patients who have a monthly income of 1500 to 5000 Ethiopian birrs were 4.38 times more likely to develop anemia compared to those who have a monthly income above 5000 birrs (AOR = 4.38, 95% CI: 1.19–16.1).

## 3. Discussion

Knowing the magnitude of hematological abnormalities in HIV/AIDS is crucial for developing effective therapeutic and preventive measures. This study assessed the prevalence of hematological abnormalities in HIV/AIDS as well as the risk factors for anemia in the Hiwot Fana Specialized University Hospital of Harar, Eastern Ethiopia.

The prevalence of anemia among HIV/AIDS patients was 16.7% (50/300) (95% CI: 12.3–21.7), the Prevalence of leucopenia was 4.33% (95% CI: 2.2–7), and the prevalence of Thrombocytopenia was 15.3% (16/300) (95% CI: 3–8.3), The identified associated factors of anemia are underweight BMI, more than 12 years duration of HIV infection, monthly income of 1500 - 5000 ethiopian birr and male sex.

This study showed a lower prevalence of anemia as compared to those studies conducted in China (47.4%),^[10]^ Mizan Tepi Ethiopia (39.8%),^[11]^ Mekelle (33.5%),^[12]^ Jinka Town (33.5%),^[13]^ Dessie town (41.3%),^[14]^ Tikuranbessa (34.6%),^[15]^ North Shewa (23.2%),^[16]^ East Africa (25.35%),^[17]^ Tanzania (40.46%),^[18]^ Southern Ethiopia (38.6%),^[19]^ Gedio Zone (34.8%)^[20]^ and East Africa meta-analysis (36.72%).^[17]^ lower prevalence of anemia in current study might be due to ART is initiated for all HIV-positive patients as early as possible and the efficacy of HAART in reducing HIV-associated anemia also, only a small no of study participants in the current study (16%) use zidovudine drugs which are known to cause anemia.^[21]^

The prevalence of leucopenia in this study was 4.33% (95% CI: 2.2–7). This finding of this study was in line with what was found in Ghana (6%).^[22]^ However, the prevalence is lower compared to studies from North Shewa (13.8%),^[16]^ Debere Tabor (34.2%),^[23]^ Jimma (12.3%),^[24]^ Nigeria (24%)^[25]^ and India (18.33%).^[7]^ The observed difference in the prevalence might be due to variations in study populations, clinical conditions, study design methods, and leukopenia definition.

In the current study the prevalence of thrombocytopenia was 5.3% (2.8–8%) This finding is in line with studies done at Debere Tabor (8.3%),^[23]^Tikuranbesa (5.7%)^[26]^ and Gondar (5.9%).^[20]^ On the other hand, the prevalence of thrombocytopenia in this study was lower than studies done in Northeastern Ethiopia (14.7%) (Talargia and Getacher, 2021), Ambo (13%),^[27]^ Cameron (6.9%),^[28]^ Uganda (13.0%),^[29]^ Ghana (18.5%),^[22]^ Nigeria (24%),^[25]^ India (15.83%) (Bhardwaj et al, 2020) and global meta-analysis (11.64%).^[30]^ This difference could be due to differences in the study population., treatment regimen, WHO stage of the participants, and thrombocytopenia definition.

The current study described that anemia among HIV/AIDS patients was significantly associated with the sex of the patient (AOR = 2.71, CI: 1.35–5.44). Thus, males are 2.71 times more likely to develop anemia compared to females, which is compatible with the findings of other studies from Arba Minch^[31]^, Debre Markos^[32]^ and Addis Ababa.^[33]^ More alcohol consumption among males than females might subsidize this. As males consume more alcohol, the rate of vitamin B12 absorption into the circulatory system becomes lower which may result in anemia. Also, khat chewing is very common in the area. More khat chewing among males than females might contribute to anemia, particularly through its impact on appetite, nutrient absorption, and potential effects on gastrointestinal and metabolic health.^[34,35]^ In contrast, other studies from China^[36]^ and Southern Ethiopia^[37]^ found females to be at more risk compared to males. The intended reason is factors like menses, pregnancy, and lactation.

The current study described that anemia among HIV/AIDS was significantly associated with BMI. As a result, the likelihood of anemia among HIV/AIDS. Patients who have a BMI category of underweight are 3.684 times more likely to develop anemia. (AOR = 3.94, 95% CI: 1.08–14.36). This is in line with studies from Debere Markos,^[32]^ South Ethiopia,^[19]^ Debere Berehan,^[38]^ and Kebata Tembaro Zone.^[37]^ This might be because being underweight can be a sign of a nutritional deficit, such as iron, folate, or cobalamin, which may cause impaired hemopoiesis and anemia, making the observed link biologically credible.

The current study described that anemia among HIV/AIDS was significantly associated with duration of HIV infection (AOR = 2.77, 95% CI: 1.43–5.39). Patients who have lived with HIV for more than 12 years were 2.77 times more prone to anemia than those who lived with HIV for <12 years. This is incongruent with studies from Sawela general hospital,^[19]^ Southwest Ethiopia,^[39]^ Welayta Sodo University Hospital^[40]^ and Malawi.^[41]^ This is due to the fact that longer standing HIV infections are associated with more advanced disease stages, lower cluster of differentiation 4 counts, and potentially more opportunistic infections all of which are known risk factors for developing anemia.

The current study described that anemia among HIV/AIDS was significantly associated with low monthly income (AOR = 4.38,95% CI: 1.19–16.09). Patients who have a monthly income of 1500 to 5000 birr are 4.38 times more prone to anemia compared to those who have a monthly income ≥ 5000 birr. This is compatible with the study from Mekele.^[12]^ This is due to factors like poor nutrition, lack of access to healthcare services and inability to afford nutritious food.

## 4. Limitation of the study

As the study is cross sectional its unable establish a temporal sequence between exposure and outcome. Also, this study didn’t collect information on nutritional status of study participants.

## 5. Conclusion and recommendation

Of the total participant’s study subjects 16.7%, 4.33%, and 5.3% were found to have anemia, leukopenia, and thrombocytopenia respectively. BMI, duration of HIV, sex and monthly income were significantly associated with the prevalence of anemia. Thus, early detection and intervention among, males, patients having BMI < 18.5 kg/m^2^, patients having low monthly income and patients who lived with HIV for more than 12 years is vital to reduce the magnitude of anemia and its consequences. Also, its better if future studies assess nutritional status of HIV/AIDS patients to better understand its effect on hematological parameters.

## Acknowledgments

We are thankful to the study participants for their voluntary participation, data collectors and staffs of HFCSH for their time and cooperation.

## Author contributions

**Conceptualization**: Beza Sileshi.

**Data curation**: Beza Sileshi, Kabtamu Gemechu.

**Formal analysis**: Beza Sileshi.

**Investigation**: Beza Sileshi.

**Methodology**: Beza Sileshi, Kabtamu Gemechu, Shambel Mekonnen, Mesay Arkew.

**Project administration**: Beza Sileshi.

**Resources**: Beza Sileshi.

**Software**: Beza Sileshi, Gada Diba, Shambel Mekonnen.

**Supervision**: Beza Sileshi, Haftu Asmerom, Mesay Arkew.

**Validation**: Beza Sileshi, Haftu Asmerom, Mesay Arkew.

**Visualization**: Beza Sileshi.

**Writing – original draft**: Beza Sileshi.

**Writing – review & editing**: Beza Sileshi.
